# 5-OH-TMT mitigates colitis through HTRA2 binding–mediated activation of the Dectin-1 signaling pathway

**DOI:** 10.1038/s41401-026-01782-0

**Published:** 2026-04-02

**Authors:** Chun-xiu Xiao, Wen-yuan Wu, Shi-cong Li, Mei-ling Dong, Yu-hang Bian, Meng Yu, Xiang Lv, Wei-guo Chen, Min Hong, Jing Zhou, Yang Sun, Hong-yue Ma, Yu-yu Zhu

**Affiliations:** 1 Jiangsu Collaborative Innovation Center of Chinese Medicinal Resources Industrialization, and Jiangsu Key Laboratory for High Technology Research of TCM Formulae, College of Pharmacy, Nanjing University of Chinese Medicine, Nanjing, 210046 China; 2 State Key Laboratory of Pharmaceutical Biotechnology and Department of Rheumatology and Immunology, Nanjing Drum Tower Hospital, the Affiliated Hospital of Nanjing University Medical School, School of Life Sciences, Nanjing University, Nanjing, 210008 Jiangsu China; 3 Jiangsu Key Laboratory of New Drug Research and Clinical Pharmacy, Xuzhou Medical University, Xuzhou, 221004 China

**Keywords:** inflammatory bowel disease, 5-OH-TMT, HTRA2, Dectin-1, intestinal barrier

## Abstract

Inflammatory bowel disease (IBD) is a debilitating condition driven by the dual pathologies of chronic inflammation and impaired intestinal barrier function. A significant clinical need exists for therapies that can effectively target both issues simultaneously. In this study, we investigated the therapeutic potential and mechanism of 5-hydroxy-*N,N,N*-trimethyltryptamine (5-OH-TMT), a quaternary ammonium salt derivative of bufotenine. We demonstrate that oral administration of 5-OH-TMT significantly ameliorates disease in two distinct murine models of experimental colitis (dextran sulfate sodium-induced and 2,4,6-trinitrobenzene sulfonic acid-induced colitis). The 5-OH-TMT treatment markedly improved clinical symptoms, potently suppressed pro-inflammatory cytokine production, and promoted a vital restoration of intestinal barrier integrity. Further exploration of the molecular basis of action of 5-OH-TMT using an unbiased proteomic screen revealed that 5-OH-TMT directly binds to and inhibits the mitochondrial serine protease high-temperature requirement A2 (HTRA2) protein. Additional mechanistic studies demonstrated that this inhibition of HTRA2 activates the Dectin-1/CARD9 signaling pathway, a key axis in mucosal defense. Subsequent work confirmed that siRNA-mediated silencing of HTRA2 could phenocopy the drug’s effects, including the suppression of pro-inflammatory NF-κB phosphorylation. In conclusion, our findings establish that 5-OH-TMT mitigates colitis through a newly identified mechanism involving direct HTRA2 inhibition. This inhibition unleashes a protective Dectin-1-dependent program that both suppresses inflammation and restores barrier function. This work identifies the HTRA2-Dectin-1 axis as a promising new therapeutic target for IBD.

**Figure:**
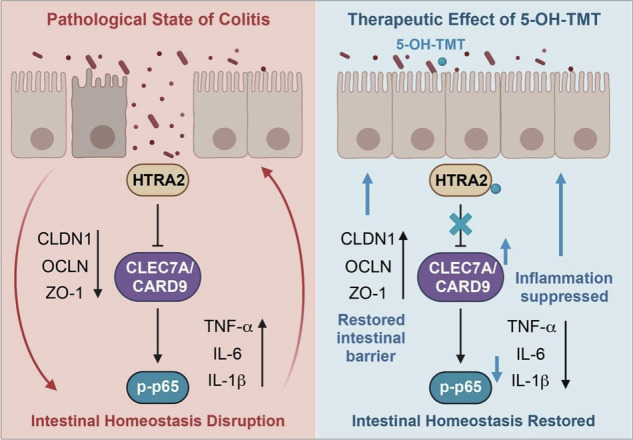
5-OH-TMT mitigates colitis through HTRA2 binding–mediated activation of the Dectin-1 signaling pathway. This figure is created with biorender.com.

5-OH-TMT mitigates colitis through HTRA2 binding–mediated activation of the Dectin-1 signaling pathway. This figure is created with biorender.com.

## Introduction

Inflammatory bowel disease (IBD), an ailment that encompasses both Crohn’s disease and ulcerative colitis, is characterized by a dysregulated mucosal immune response that produces a chronic and relapsing inflammatory disorder of the gastrointestinal tract [1]. The pathogenesis of IBD is multifactorial and driven by a complex interplay of genetic susceptibility, environmental factors, and an aberrant host response to the gut microbiota [2]. Two interconnected pathological hallmarks are central to IBD progression: a relentless pro-inflammatory cytokine cascade [3, 4], dominated by mediators such as TNF-*α*, IL-6, and IL-1*β*, and a profound defect in the intestinal epithelial barrier [5–7]. The barrier dysfunction, which is characterized by the loss of tight junction integrity, increases intestinal permeability, thereby allowing luminal antigens to penetrate the mucosa and perpetuate a vicious cycle of inflammation [8, 9]. Current IBD management includes the use of biologic therapies targeting key cytokines, and while this approach has revolutionized disease management, a significant number of patients exhibit primary nonresponse, secondary loss of efficacy, or adverse side effects [10–14]. These recalcitrant responses to treatment underscore the ongoing need for novel therapeutic agents with distinct mechanisms of action for the treatment of IBD.

An ideal IBD therapeutic strategy would suppress inflammation while also actively promoting mucosal healing and the restoration of barrier function, thus requiring the identification of molecular targets that can simultaneously regulate all three processes. One potential molecular target is high-temperature requirement A2 (HTRA2), also known as Omi. HTRA2 is a mitochondrial serine protease that plays a critical role in mitochondrial quality control and has well-documented proapoptotic functions [15–17]. Interestingly, emerging evidence now supports a broader role for HTRA2 in promoting cellular stress responses and inflammation. At present, no specific function for HTRA2 has been identified in the context of intestinal inflammation, and its potential as a therapeutic target for IBD remains unexplored. In the present study, we hypothesized that HRTA2 could be a promoter of inflammation in IBD and could therefore be targeted using anti-inflammatory drugs, such as tryptamines.

Tryptamines are a class of naturally occurring indolealkylamines. One interesting group of tryptamines comprises, bufotenine and its congeners, which are found in the toxic secretions of toad species such as *Bufo bufo gargarizans* Cantor and *Bufo melanostictus* Schneider [18]. Previous studies have highlighted the potent anti-inflammatory effects of bufotenine in various disease models [19–21]; however, its direct molecular targets and the precise mechanisms underlying its therapeutic action remain poorly understood.

The aim of the present study was to investigate the potential effectiveness of bufotenine as a treatment for IBD. However, recognizing the challenges of direct isolation of bufotenine from natural sources is challenging, and the resulting low yields, we opted instead to synthesize a quaternary ammonium salt bufotenine derivative, 5-hydroxy-*N,N,N*-trimethyltryptamine (5-OH-TMT) [22], hypothesizing that it could represent a promising therapeutic candidate for inflammatory bowel disease (IBD). We systematically investigated the therapeutic efficacy and underlying molecular mechanism of 5-OH-TMT in two mouse experimental colitis models. Our findings confirm that oral administration of 5-OH-TMT potently ameliorates colitis in both DSS- and TNBS-induced mouse models by suppressing proinflammatory cytokine production and restoring intestinal barrier integrity.

Specifically, our use of a combination of unbiased target identification and biophysical validation assays conclusively identify HTRA2 as a direct and specific molecular target of 5-OH-TMT. Mechanistically, we show that the 5-OH-TMT-mediated inhibition of HTRA2 releases a brake on the C-type lectin receptor signaling pathway to activate a Dectin-1/CARD9-dependent mucosal defense program. A particularly notable finding is that genetic silencing of *HTRA2* phenocopies the drug effects, thereby confirming that HTRA2 inhibition is sufficient to suppress NF-κB signaling and enhance epithelial barrier function. Our comprehensive approach has uncovered a novel therapeutic axis in which the direct inhibition of HTRA2 orchestrates a dual protective response. The effectiveness of 5-OH-TMT at inhibiting HTRA2 effects positions this drug as a promising agent for the treatment of IBD.

## Materials and methods

### Synthesis of 5-OH-TMT

5-OH-TMT was synthesized using the previous method [22]. The synthesis of 5-OH-TMT commenced with the acylation of 5-benzyloxyindole at the C3-position using oxalyl chloride. The resulting acyl chloride was subsequently treated with dimethylamine hydrochloride to yield the *N, N*-dimethyl indol-3-ylglyoxylamide intermediate. This intermediate was then reduced with lithium aluminum hydride (LiAlH_4_), which converted both carbonyl groups to form the tryptamine side chain. Finally, the benzyl protecting group was removed via catalytic hydrogenation over palladium on carbon (Pd/C) to afford the final product, 5-OH-TMT.

### Animal

C57BL/6 mice (male, 6–8 weeks old) were obtained from GemPharmatech Co., Ltd. (Nanjing, China) and were co-housed for at least one week prior to experiments. Animals were maintained in a specific pathogen-free facility under a 12-h light/dark cycle at a controlled temperature (22 ± 2 °C), with *ad libitum* access to standard chow and water. All animal experiments were conducted in strict accordance with the NIH Guide for the Care and Use of Laboratory Animals and were reviewed and approved by the Institutional Animal Care and Use Committee (IACUC) of Nanjing University of Chinese Medicine (Approval No. 202304A032).

### Dextran sulfate sodium (DSS)-induced colitis

Acute colitis was induced by administering 3% (w/v) DSS (MW 40–50 kDa; MP Biomedicals) in the drinking water for 7 consecutive days, followed by a 3-day recovery period with regular water. On day 10, C57BL/6 male mice were euthanised, and colons were rapidly excised. Colon length was measured from the caecal-colonic junction to the anal verge as a macroscopic indicator of inflammation. Distal colon segments were then procured for histological analysis (H&E and immunofluorescence), protein quantification (ELISA and western blotting), and gene expression analysis (qRT-PCR).

### 2,4,6-trinitrobenzene sulfonic acid (TNBS)-induced colitis

Colitis was induced using a previously described two-step protocol [23]. On day 1, C57BL/6 male mice were epicutaneously sensitized by applying 200 µL of 1% TNBS in an acetone and olive oil vehicle (1:1 *v*/*v*) to a shaved dorsal surface. On day 7, mice were anaesthetized with isoflurane, and 100 µL of 2.5% TNBS in 50% ethanol was instilled intrarectally via a 3.5 F catheter. Following induction, mice were randomised into treatment groups. The sham and TNBS-vehicle groups received 0.9% saline daily via oral gavage from day 7 to day 9. Treatment groups received either 15 mg/kg or 30 mg/kg 5-OH-TMT, administered by oral gavage over the same period. On day 10, mice were euthanised, and colons were harvested and analysed as described for the DSS model.

### Quantitative Real-Time PCR (qRT-PCR)

Total RNA was extracted from frozen mouse colon tissues or NCM460 cells using the RNA Isolater Total RNA Extraction Reagent (Vazyme Biotech Co., Ltd., China) according to the manufacturer’s protocol. One microgram of total RNA was subsequently reverse-transcribed into cDNA using the HiScript III RT SuperMix for qPCR (Vazyme Biotech Co., Ltd., China). qRT-PCR was performed using AceQ Universal SYBR qPCR Master Mix (Vazyme Biotech Co. Ltd, China) on a CFX100 Real-Time PCR Detection System (Bio-Rad, Hercules, CA). The thermal cycling conditions were: 95 °C for 2.5 min, followed by 35 cycles of 95 °C for 15 s and 60 °C for 30 s. A melt curve analysis was performed to verify amplicon specificity. Relative mRNA expression was calculated using the 2^-ΔΔCt^ method and normalized to the housekeeping genes *Gapdh* (for mouse) or *ACTIN* (for human). All primer sequences are listed in Supplementary Table S1.

### Enzyme-Linked Immuno Sorbent (ELISA) assay

Concentrations of tumor necrosis factor-*α* (TNF-*α*) and interleukin-1*β* (IL-1*β*) in mouse colon homogenates and serum were quantified using commercial ELISA kits (Multi Sciences; TNF-*α*, Cat# EK282; IL-1*β*, Cat# EK201B) according to the manufacturer’s instructions.

### Histological analysis

Formalin-fixed, paraffin-embedded mouse colon tissues were sectioned at 5 µm. For morphological assessment, sections were stained with haematoxylin and eosin (H&E). For immunofluorescence (IF), sections underwent heat-induced epitope retrieval in sodium citrate buffer, were permeabilised with 0.5% Triton X-100, and blocked with 5% normal goat serum. Sections were then incubated overnight at 4 °C with the following primary antibodies (all diluted 1:100): anti-CLDN1 (Abmart, PA2349F), anti-OCLN (Abmart, TD7504F), anti-ZO1 (Abmart, TA5145F), anti-CLEC7A (Abmart, PC2455S), anti-CARD9 (Abmart, TU721343S), anti-E-cadherin (HUABIO, EM0502), or anti-phospho-p65 (Abmart, TP56372F). Detection was performed using Alexa Fluor-conjugated secondary antibodies (1:200; Absin), and nuclei were counterstained with DAPI.

### Cell culture

The human intestinal epithelial cell line NCM460 was obtained from Fenghui (Hunan, China). Cells were cultured in Dulbecco’s Modified Eagle’s Medium (DMEM; Gibco, USA) supplemented with 10% fetal bovine serum (FBS; EvaCell, USA) and 1% penicillin-streptomycin solution (Beyotime, China). The cell cultures were maintained at 37 °C in a humidified atmosphere containing 5% CO_2_.

### Target-responsive accessibility profiling (TRAP)

TRAP was employed to identify protein targets of 5-OH-TMT by quantifying ligand-induced changes in lysine accessibility across the proteome, as previously described [24]. Briefly, NCM460 cells were treated with 3 µM 5-OH-TMT or vehicle (ddH₂O) for 2 h. Cells were then lysed, and accessible lysine residues were covalently labelled via dimethylation. The proteome was precipitated, resolubilised in 8 M urea, reduced with DTT, alkylated with iodoacetamide, and digested with trypsin. Peptides were desalted and analysed by nanoLC-MS/MS on a SYNAPT G2-Si Q-TOF mass spectrometer (Waters) in data-dependent acquisition mode. Raw data were analysed using PEAKS Studio 8.5. Peptides were considered significant hits if they exhibited a TRAP ratio of >2 or <0.5 and a *P*-value < 0.05, indicating 5-OH-TMT-induced changes in lysine accessibility.

### Western blotting

Protein lysates from cells or colon tissues were prepared using cell lysis buffer for Western and IP (Beyotime, China) supplemented with protease inhibitors. Protein concentrations were determined using a Bradford assay. Equal amounts of protein (20–30 µg) were resolved by SDS-PAGE, transferred to PVDF membranes (Millipore), and blocked with 5% non-fat milk in TBST. Membranes were incubated overnight at 4 °C with primary antibodies, followed by incubation with HRP-conjugated secondary antibodies. Bands were visualized using an enhanced chemiluminescence substrate system. Primary antibodies used were: anti-HTRA2 (1:2000; Proteintech, 15775-1-AP), anti-HTRA1 (1:1000; abcam, AB274322), anti-CLEC7A (1:500; Abmart, PC2455S), anti-CARD9 (1:1,000; Abmart, TU721343S), anti-phospho-p65 (1:500; Abmart, TP56372F), anti-p65 (1:5,000; Abmart, T55034F), anti-Actin (1:1,000; Abmart, M20011M), anti-Tubulin (1:10,000; Abways, AB0039) and anti-GAPDH (1:3,000; Abmart, P60037S).

### Cellular thermal shift assay (CETSA)

Target engagement was validated by CETSA [25]. NCM460 cells were incubated with 5-OH-TMT or vehicle for 2 h. After washing, cell suspensions were heated across a thermal gradient (50–72 °C, 2 °C increments) for 3 min. Lysis was induced by three freeze–thaw cycles. The soluble fraction was separated from the aggregated pellet by centrifugation (20,000 × *g*, 20 min, 4 °C). The relative abundance of soluble target protein in the supernatant at each temperature was assessed by Western blot to determine the ligand-induced thermal shift.

### Drug Affinity Responsive Target Stability (DARTS)

Direct binding was confirmed using the DARTS assay [26]. NCM460 cell lysate was incubated with 5-OH-TMT (1 µM or 3 µM) or vehicle for 1 hour at room temperature. Samples were then subjected to limited proteolysis with Pronase (Sigma-Aldrich) for 10 minutes. The reaction was terminated with loading buffer and boiling. Samples, including undigested controls, were analysed by western blot with an anti-HTRA2 or anti-HTRA1 antibody. Protection from degradation indicated direct binding.

### Molecular docking

The crystal structure of human HTRA2 was retrieved from the RCSB Protein Data Bank (PDB). Computational docking was performed using the Schrödinger Suite 2018. The HTRA2 structure was prepared using the Protein Preparation Wizard, and the 5-OH-TMT ligand structure was prepared using LigPrep. Ligand docking was performed with Glide, generating a receptor grid centered on the active site. The top-ranked pose, based on GlideScore, was selected for interaction analysis and visualized using PyMOL (v2.5.4).

### Microscale Thermophoresis (MST)

HEK293T cells were first transfected with plasmids encoding EGFP-tagged wild-type HTRA2 (EGFP-HTRA2^wt^), EGFP-HTRA2^N181A^, or EGFP-HTRA2^K395A^. After 48 hours, cells were harvested, lysed, and clarified by centrifugation. The resulting cell lysates were mixed at a 1:1 volume ratio with serial dilutions of 5-OH-TMT and incubated for 10 minutes. Samples were subsequently loaded into zero-background capillaries, and thermophoresis was quantified using a Monolith NT.LabelFree instrument (NanoTemper Technologies).

### RNA-seq

Total RNA was extracted from NCM460 cells using Trizol reagent (Vazyme Biotech Co., Ltd., China). RNA quality and integrity were confirmed using a NanoDrop ND-2000 spectrophotometer (Thermo Scientific, MA, USA) and an Agilent Bioanalyzer 2100 (Agilent Technologies, Palo Alto, California, USA), respectively. Library preparation and sequencing were performed by Novogene Co., Ltd. (Beijing, China). Differentially expressed genes (DEGs) were identified based on a fold change ≥2 and a *P*-value ≤ 0.05. The raw sequencing data have been deposited in the Gene Expression Omnibus (GEO) database under accession number GSE302465.

### RNA interference

Small interfering RNA (siRNA) targeting human *HTRA2* was synthesized by GENERAL BIOL: 1# sense: 5′-UCGCAGAUGUGGUGGAGAATT-3′, antisense: 5′-UUCUCCACCACAUCUGCGATT-3′; 2# sense: 5′-GGGCAGUGCUGUUGUUGUUTT-3', antisense: 5′-AACAACAACAGCACUGCCCTT-3′; 3# sense: 5′-GGUACAAAAUGCUGAAGAUTT-3′, antisense: 5′-AUCUUCAGCAUUUUGUACCTT-3′. siRNA transfection was performed using the Hieff Trans® Transfection Reagent (Yeasen Biotechnology (Shanghai) Co., Ltd.) according to the manufacturer’s protocol.

### Lentiviral transduction

Lentiviral particles targeting HTRA2, as well as a negative control (NC) lentivirus, were obtained from Shanghai Obio Technology Co., Ltd. (Shanghai, China). NCM460 cells were transduced with either the NC or the HTRA2-targeting lentivirus and incubated for 48 h. To establish stable cell lines, infected cells were subjected to selection with 0.5 μg·mL⁻¹ puromycin (Sigma–Aldrich) for 14 days. Overexpression efficiency was subsequently verified via immunoblotting.

### Statistical analysis

Data are presented as mean ± SEM. Statistical significance was determined using GraphPad Prism 9.0. Comparisons between two groups were performed using an unpaired, two-tailed Student’s *t*-test. Comparisons among multiple groups were performed using Tukey’s multiple-comparison test. A *P*-value < 0.05 was considered statistically significant. Significance is denoted in figures as *P < 0.05, **P < 0.01.

## Results

### 5-OH-TMT ameliorates DSS-induced colitis

**Fig. 1:**
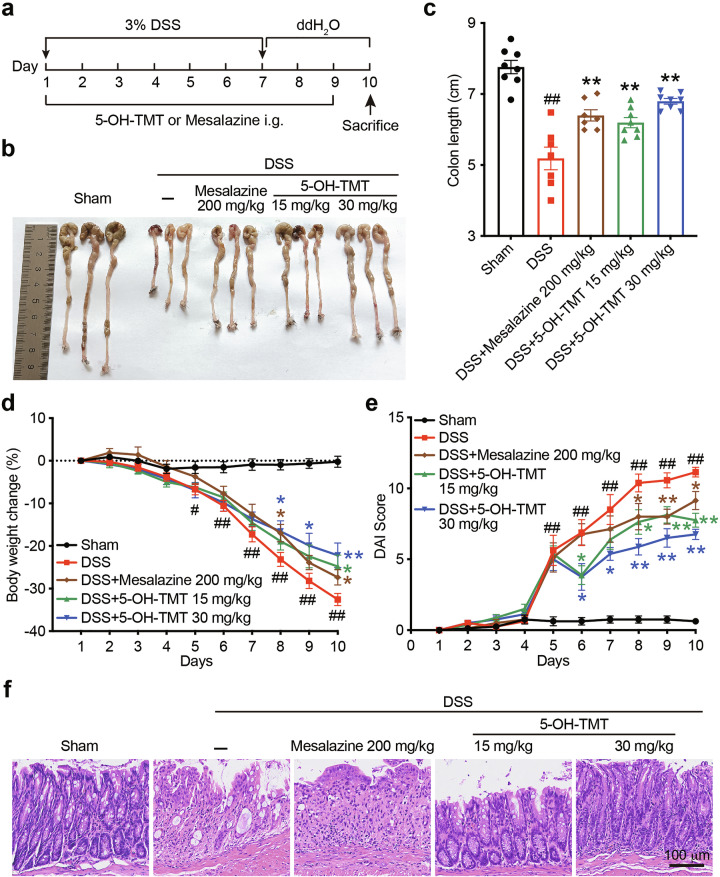
5-OH-TMT ameliorates DSS-induced colitis. **a** Schematic diagram of the animal experiment. Acute colitis was induced by administration of 3% DSS in drinking water for 7 days, followed by regular water for 3 days. Mice were treated with 5-OH-TMT (15 or 30 mg/kg, i.g.) or mesalazine (200 mg/kg, i.g.) daily from day 1 to day 9. Mice were sacrificed on day 10. **b** Representative images of colons from each group at sacrifice. **c** Quantification of colon length in each group (*n* = 8 per group). **d**, **e** The body weight change (**d**) and DAI scores (**e**) of indicated mice was measured daily over 10 days. **f** Representative histopathological images of colon tissue sections stained with H&E. Scale bar, 100 μm. Data are presented as mean ± SEM. *P* values are determined by Tukey multiple comparison test (**c**–**e**). ^##^*P* < 0.01 vs. Sham; ^#^*P* < 0.05 vs. Sham; ^**^P < 0.01 vs. DSS; ^*^P < 0.05 vs. DSS.

We evaluated the therapeutic potential of 5-OH-TMT in vivo by first establishing an acute colitis model in C57BL/6 mice using dextran sodium sulfate (DSS), according to the treatment regimen outlined in (Fig. 1a). Mice administered DSS developed severe colitis, which was macroscopically evident by a significant shortening of the colon compared with healthy controls. Oral administration of 5-OH-TMT markedly attenuated this colon shortening, indicating a protective effect on colonic structural integrity (Fig. 1b, c). Consistent with the macroscopic findings, the mice in the DSS group exhibited rapid and progressive body weight loss and a correspondingly high disease activity index (DAI) score (reflecting weight loss, stool consistency, and rectal bleeding). Treatment with 5-OH-TMT significantly mitigated the weight loss (Fig. 1d) and lowered the DAI scores (Fig. 1e).

Histological analysis of H&E–stained colon sections confirmed the protective effect of 5-OH-TMT at the tissue level. The DSS group displayed severe histopathological damage, characterized by extensive destruction of the mucosal architecture, loss of crypts, depletion of goblet cells, and massive inflammatory cell infiltration into the lamina propria. In contrast, 5-OH-TMT treatment substantially preserved the colon’s structural integrity, maintaining crypt architecture, restoring goblet cell numbers, and markedly reducing immune cell infiltration (Fig. 1f). Collectively, these data demonstrated that 5-OH-TMT effectively alleviated the clinical and pathological features of DSS-induced colitis in mice.

### 5-OH-TMT protects against TNBS-induced colitis

**Fig. 2:**
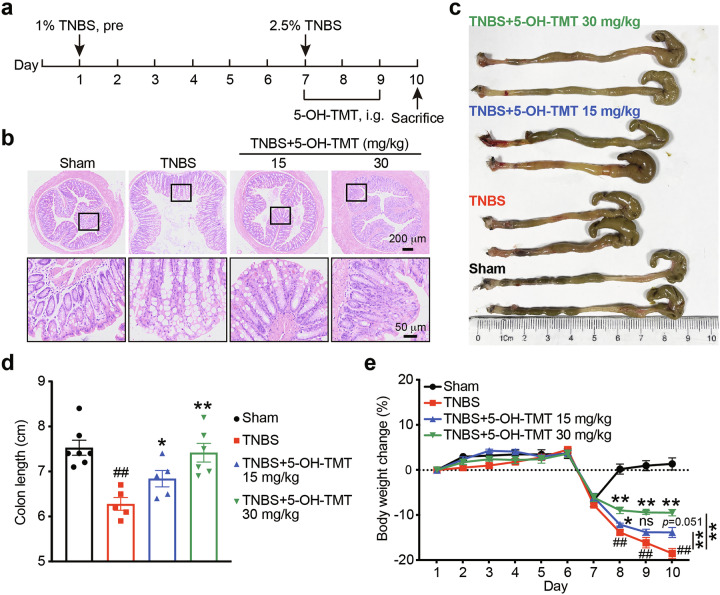
5-OH-TMT protects against TNBS-induced colitis. **a** Schematic of the animal experiment. Mice were pre-sensitized with 1% TNBS on day 1, and colitis was induced by 2.5% TNBS on day 7. 5-OH-TMT (15 or 30 mg/kg, i.g.) was administered daily from day 7 to day 9. Mice were sacrificed on day 10. **b** Representative H&E-stained sections of colon tissues from each group (upper panels: scale bar, 200 μm; lower panels: magnified insets, scale bar, 50 μm). **c** Representative images of colons collected from each group at sacrifice. **d** Quantification of colon length (*n* = 5-7 per group). **e** Body weight change (%) during the experiment. Data are presented as mean ± SEM. *P* values are determined by Tukey multiple comparison test (**d**, **e**). ^##^*P* < 0.01 vs. Sham; ^**^P < 0.01, ^*^P < 0.05 vs. TNBS.

We further validated the therapeutic efficacy of 5-OH-TMT in a mechanistically distinct model by assessing the 5-OH-TMT effects in a T-cell–dependent model of TNBS-induced colitis (Fig. 2a). Histopathological analysis of H&E-stained colon sections revealed that 5-OH-TMT treatment conferred significant tissue protection. While mice in the TNBS group displayed severe colon damage, characterized by extensive destruction of the mucosal architecture, loss of crypts, and a dense inflammatory infiltrate, the mice treated with 5-OH-TMT showed a marked amelioration of these pathological features. In the 5-OH-TMT-treated group, the colonic mucosal structure was substantially more intact, crypt damage was diminished, and immune cell infiltration was significantly reduced (Fig. 2b).

Macroscopic examination of the excised colons corroborated these histological findings. The colons of the TNBS group were visibly inflamed, exhibiting edematous wall thickening and a significant reduction in length. In contrast, oral administration of 5-OH-TMT effectively counteracted these inflammatory changes, resulting in a significant preservation of colon length compared with the TNBS group (Fig. 2c, d). The protective effects of 5-OH-TMT were also reflected in systemic clinical signs. Notably, the TNBS group mice experienced a progressive and significant loss of body weight, but this weight loss was significantly mitigated in mice that had received the 5-OH-TMT treatment (Fig. 2e). Collectively, these findings confirmed that 5-OH-TMT exerted potent anti-inflammatory and tissue-protective effects, reinforcing its therapeutic potential as a treatment for IBD.

### 5-OH-TMT modulates the inflammatory cytokine network in experimental colitis

**Fig. 3:**
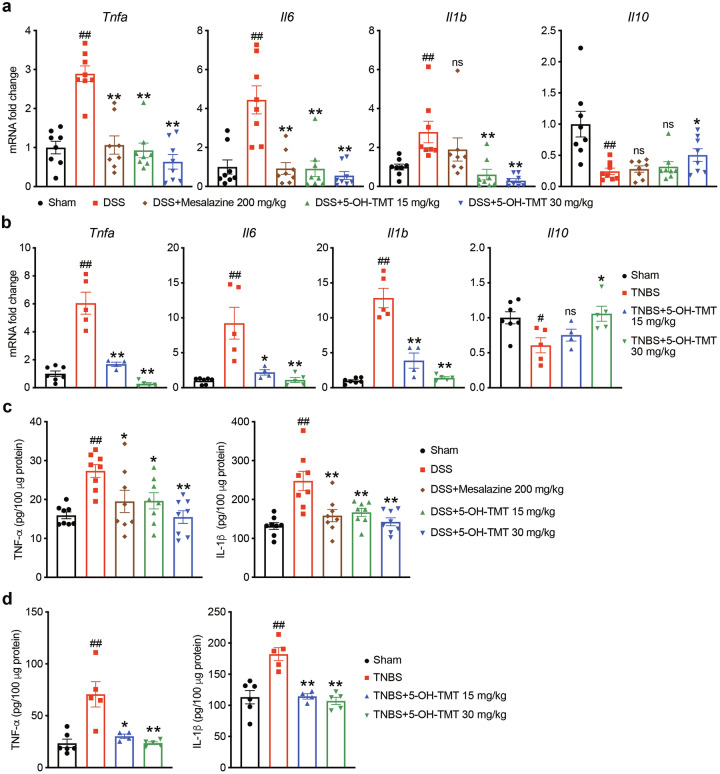
5-OH-TMT modulates the inflammatory cytokine network in experimental colitis. **a**, **b** Quantitative PCR analysis of mRNA encoding colitis-related inflammatory cytokines in the colon of DSS-induced (**a**) or TNBS-indued (**b**) mice treated with indicated dose of 5-OH-TMT or vehicle for 10 days (*n* = 4–8 per group). Results were normalized to *Gapdh* expression. **c**, **d** ELISA quantification of TNF-*α* and IL-1*β* protein levels in the colon of DSS-induced (**c**) or TNBS-indued (**d**) mice treated with indicated dose of 5-OH-TMT or vehicle for 10 days (*n* = 4–8 per group). Data are presented as mean ± SEM. *P* values are determined by Tukey multiple comparison test. ^##^*P* < 0.01, ^#^*P* < 0.05 vs. Sham; ^**^P < 0.01, ^*^P < 0.05 vs. DSS or TNBS group. ns, not significant.

We elucidated the anti-inflammatory mechanism of 5-OH-TMT by assessing its impact on cytokine expression in both the DSS- and TNBS-induced mouse colitis models. qRT-PCR analysis revealed a significant upregulation of the colonic expression of the pro-inflammatory cytokine genes *Tnfa*, *Il6*, and *Il1b* in both colitis models, whereas oral administration of 5-OH-TMT markedly suppressed the transcription of these genes in both models. Conversely, treatment with 30 mg/kg 5-OH-TMT significantly increased the expression of the anti-inflammatory cytokine *Il10* (Fig. 3a, b).

We determined whether these transcriptional changes were translated at the protein level by quantifying the cytokine concentrations in colonic homogenates using ELISA. Consistent with the mRNA data, the protein levels of TNF-*α* and IL-1*β* were significantly elevated in the colons of the DSS- and TNBS-induced colitis models, whereas 5-OH-TMT treatment robustly inhibited the production of both cytokines in both models (Fig. 3c, d), thereby confirming the potent local anti-inflammatory activity of 5-OH-TMT.

We then examined whether 5-OH-TMT also modulated systemic inflammation by analyzing the serum from mice with DSS-induced colitis. The circulating levels of TNF-*α* and IL-1*β* were significantly increased following DSS administration, but this systemic inflammatory response was potently suppressed by 5-OH-TMT treatment (Fig. S1). Collectively, these data demonstrated that a key therapeutic mechanism underlying the effects of 5-OH-TMT in experimental colitis was the potent suppression of both local and systemic pro-inflammatory cytokine networks.

### 5-OH-TMT restores intestinal epithelial barrier integrity in experimental colitis

**Fig. 4:**
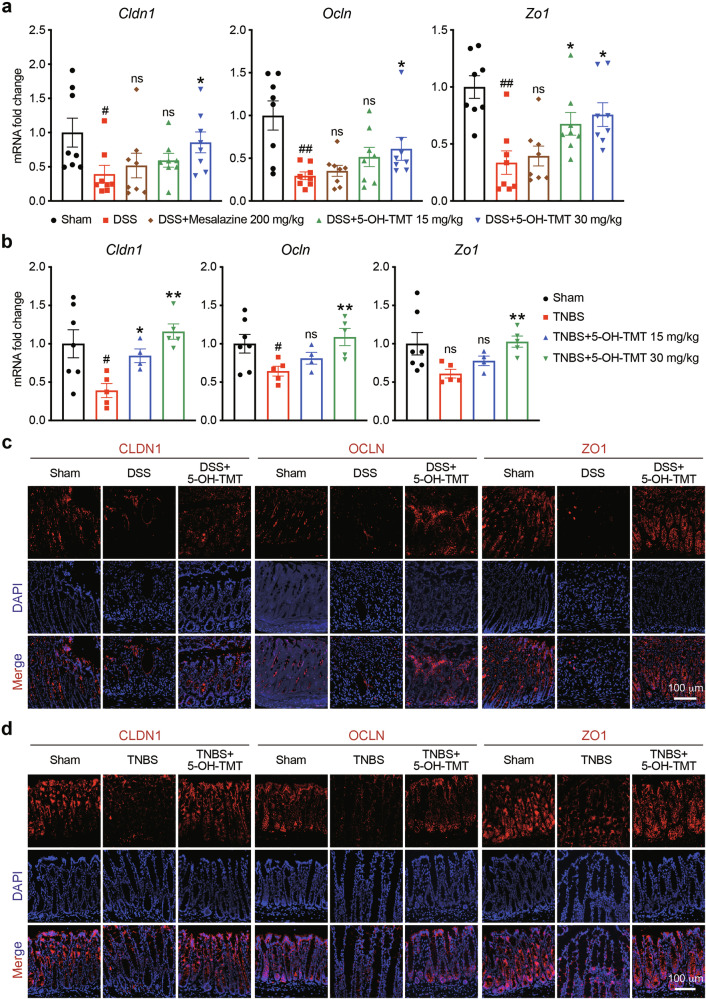
5-OH-TMT restores intestinal epithelial barrier integrity in experimental colitis. **a**, **b** Quantitative PCR analysis of mRNA encoding colitis-related tight junction protein in the colon of DSS-induced (**a**) or TNBS-indued (**b**) mice treated with indicated dose of 5-OH-TMT or vehicle for 10 days. Results were normalized to *Gapdh* expression. **c**, **d** Representative immunofluorescence staining of CLDN1, OCLN, and ZO1 (*red*), with DAPI (*blue*) counterstaining, in colon tissue from DSS-induced (**c**) or TNBS-indued (**d**) mice treated with indicated dose of 5-OH-TMT or vehicle for 10 days. Scale bar, 100 μm. Data are shown as mean ± SEM. *P* values are determined by Tukey multiple comparison test (**a**, **b**). ^##^*P* < 0.01, ^#^*P* < 0.05 vs. Sham; ^**^P < 0.01, ^*^P < 0.05 vs. DSS or TNBS group. ns, not significant.

Impairment of the intestinal epithelial barrier is a key pathophysiological feature of IBD [27, 28]. Therefore, we investigated whether the therapeutic effects of 5-OH-TMT involved the preservation of barrier function. We first assessed the expression of genes encoding key tight junction (TJ) proteins. Quantitative RT-PCR analysis showed significant downregulation of the colonic expression of *Cldn1* (claudin-1), *Ocln* (occludin), and *Tjp1* (tight junction protein 1, also known as ZO-1) in both DSS- and TNBS-induced colitis, whereas 5-OH-TMT treatment significantly restored the expression of all three TJ genes in both models (Fig. 4a, b).

We confirmed these findings at the protein level by performing immunofluorescence staining for CLDN1, OCLN, and ZO-1. Colon sections from vehicle-treated mice with colitis showed marked reductions in the expression of these TJ proteins and severe disruption of their linear organization at the apical intercellular junctions of epithelial cells. In stark contrast, 5-OH-TMT treatment enhanced the overall expression of CLDN1, OCLN, and ZO-1 while also restoring their proper localization to the cell–cell junctions of the colon epithelium (Fig. 4c, d). Taken together, these data demonstrated that 5-OH-TMT reinforced the intestinal epithelial barrier by promoting the expression and correct assembly of key tight junction proteins. This restoration of barrier integrity is a critical component of the protective effects of 5-OH-TMT against colon damage in experimental colitis.

We also further investigated the impact of 5-OH-TMT on mucosal immunity by assessing the expression of antimicrobial peptides in the colon. 5-OH-TMT treatment markedly increased the mRNA levels of *Defb1*, *S100a8*, and *S100a9* in the DSS- and TNBS-induced colitis models (Fig. S2), suggesting an enhancement of epithelial antimicrobial defenses.

### 5-OH-TMT directly targets HTRA2

**Fig. 5:**
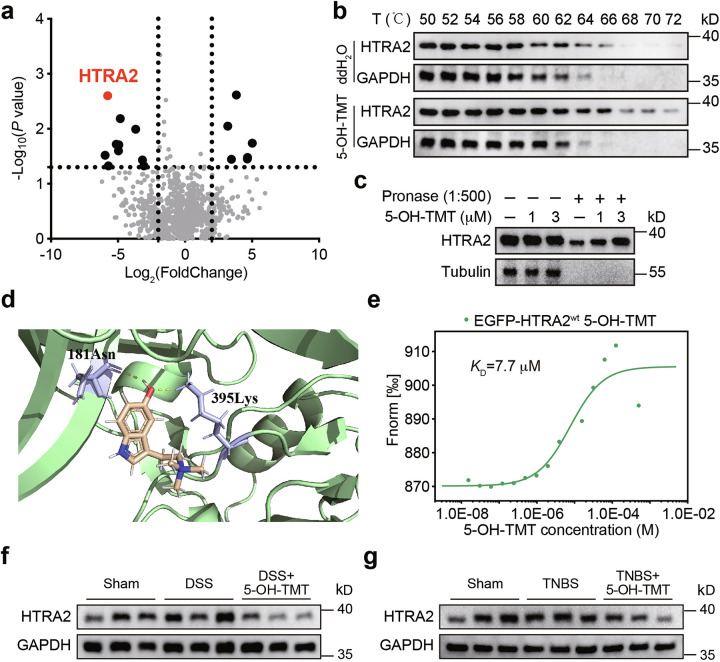
5-OH-TMT directly targets HTRA2. **a** 5-OH-TMT binding proteins using target-responsive accessibility profiling (TRAP) experiment. **b** NCM460 cells were incubated with 5-OH-TMT or ddH_2_O for 2 h, and CETSA analyzed the thermal stabilization of HTRA2 protein at different temperatures. **c** 5-OH-TMT enhanced HTRA2 resistance to proteases, which was investigated by DARTS. **d** Molecular docking model depicting the interaction of 5-OH-TMT with HTRA2. **e** The interaction of 5-OH-TMT with HTRA2 was measured by MST. **f**, **g** Western blot analysis of HTRA2 protein expression in colon tissues from DSS (**f**) and TNBS (**g**) colitis models treated with or without 30 mg/kg 5-OH-TMT.

We explored the molecular mechanisms underlying the protective effects of 5-OH-TMT by first determining a noncytotoxic concentration for mechanistic studies in NCM460 cells. We selected 3 µM based on our CCK-8 assay results (Fig. S3). We then used a target-responsive accessibility profiling (TRAP) assay to identify the direct molecular targets of 5-OH-TMT. This screen revealed that the mitochondrial serine protease, high-temperature requirement A2 (HTRA2), showed a significant change in binding accessibility upon 5-OH-TMT treatment, indicating HTRA2 as a primary candidate (Fig. 5a).

We then validated this interaction using orthogonal biophysical assays. A cellular thermal shift assay (CETSA) confirmed direct target engagement in situ, demonstrating that 5-OH-TMT significantly enhanced the thermal stability of the endogenous HTRA2 protein (Fig. 5b). Drug affinity responsive target stability (DARTS) assays also showed that 5-OH-TMT protected native HTRA2 from proteolytic degradation, thereby confirming a direct physical interaction (Fig. 5c). Notably, this binding was selective, as 5-OH-TMT did not protect the closely related HTRA1 homolog from degradation, ruling out off-target effects on this protein (Fig. S4).

We defined the binding interface using molecular docking simulations, which predicted that 5-OH-TMT fits within the catalytic pocket of HTRA2, forming key hydrogen bonds with residues Asn181 and Lys395 (Figs. 5d and S5). Therefore, we constructed EGFP-tagged plasmids for wild-type (WT) HTRA2 and two catalytic site point mutants (N181A and K395A). Using microscale thermophoresis (MST), we quantitatively assessed the binding affinity and determined that 5-OH-TMT bound to WT HTRA2 with a *K*_D_ of 7.7 µM (Fig. 5e).

The binding affinity was profoundly weakened by mutations at the predicted site, decreasing by more than an order of magnitude, with *K*_D_ values of 80.8 µM for the N181A mutant and 89.3 µM for the K395A mutant (Fig. S6). This ~10-fold reduction in binding affinity upon mutation of key catalytic residues demonstrated that the interaction between 5-OH-TMT and HTRA2 was specific. Deeper analysis of the TRAP data pinpointed a peptide within the HTRA2 protease domain (amino acids 234–247) whose accessibility was directly altered by 5-OH-TMT binding, suggesting that 5-OH-TMT affected the substrate-binding or catalytic regions (Fig. S7).

We also observed a significant downregulation of HTRA2 protein expression, in the colon after treatment of colitis mice with 5-OH-TMT (Fig. 5f, g). Together, these data identified HTRA2 as a direct and specific molecular target of 5-OH-TMT both in vitro and in vivo.

### 5-OH-TMT activates the Dectin-1 signaling pathway through HTRA2

**Fig. 6:**
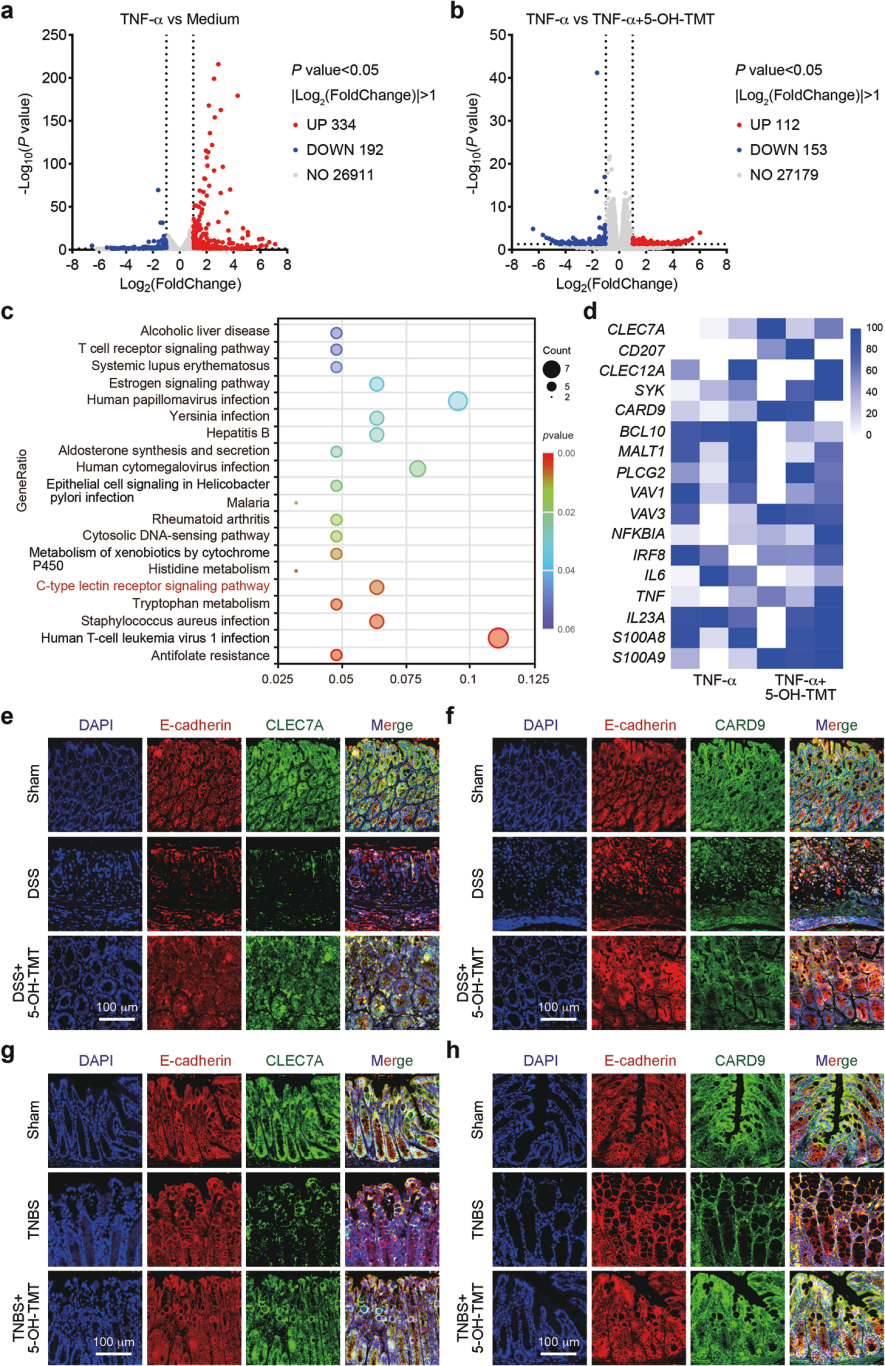
5-OH-TMT activates the Dectin-1 signaling pathway through HTRA2. **a**, **b** Volcano plots showing differentially expressed genes in NCM460 cells treated with TNF-*α* versus medium (**a**), and TNF-*α* plus 5-OH-TMT versus TNF-*α* (**b**) ( | Log_2_(Fold Change)|>1, *P* < 0.05; red: upregulated, blue: downregulated) (*n* = 3/group). **c** KEGG pathway analysis was performed on the differentially expressed genes, highlighting C-type lectin receptor signaling. **d** Heatmap of representative genes in the C-type lectin receptor signaling pathway, showing relative expression in TNF-*α* and TNF-*α* plus 5-OH-TMT groups. **e**, **g** Colon tissue sections from DSS-induced (**e**) or TNBS-induced (**g**) mice treated with 30 mg/kg 5-OH-TMT or vehicle were immune-stained for CLEC7A (*green*) together with a marker of epithelial cells (E-cadherin, *red*) prior to analysis by confocal microscopy. Scale bars, 100 μm. **f**, **h** Colon tissue sections from DSS-induced (**f**) or TNBS-induced (**h**) mice treated with 30 mg/kg 5-OH-TMT or vehicle were immune-stained for CARD9 (*green*) together with a marker of epithelial cells (E-cadherin, red) prior to analysis by confocal microscopy. Scale bars, 100 μm.

We explored potential connections between the inhibition of HTRA2 and the observed therapeutic effects by identifying the downstream signaling pathways involved. RNA sequencing of TNF-*α*-stimulated NCM460 intestinal epithelial cells revealed that 5-OH-TMT treatment significantly altered the inflammatory transcriptome. KEGG analysis identified a significant enrichment of the C-type lectin receptor (CLR) signaling pathway, with significant upregulation of key components, including *CLEC7A* (Dectin-1), *CARD9*, and *SYK* (Figs. 6a–d and S8, 9). Subsequent in vivo experiments in DSS-induced and TNBS-induced mouse colitis models revealed consistent activation of this axis by 5-OH-TMT, as treatment robustly increased the protein expression of both Dectin-1 and its downstream adaptor CARD9 in the colonic epithelium (Figs. 6e–h and S10).

### HTRA2 knockdown regulates C-type lectin receptor signaling

**Fig. 7:**
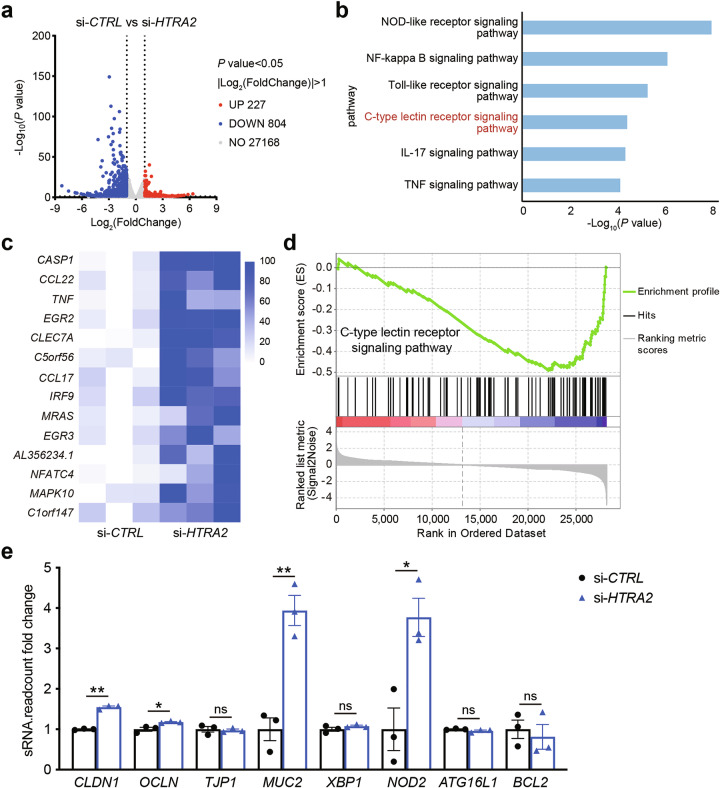
HTRA2 knockdown regulates C-type lectin receptor signaling. **a** Volcano plot showing differentially expressed genes in NCM460 cells transfected with si-*HTRA2* versus si-*CTRL* (|Log₂(Fold Change)|>1, *P* < 0.05; red: upregulated, blue: downregulated). **b** KEGG pathway enrichment analysis of differentially expressed genes, highlighting significant enrichment of C-type lectin receptor signaling pathway. **c** Heatmap showing relative expression of representative genes involved in C-type lectin receptor signaling in si-*CTRL* and si-*HTRA2* groups. **d** Gene set enrichment analysis (GSEA) showing negative enrichment of the C-type lectin receptor signaling pathway in si-*HTRA2* NCM460 cells. **e** Quantification of key epithelial and immune-related gene expression by sRNA readcount in si-*CTRL* and si-*HTRA2* groups (*n* = 3 per group). Data are presented as mean ± SEM. *P* values are determined by two-tailed Student’s *t*-test (**e**). ^**^P < 0.01, ^*^P < 0.05, ns not significant.

Having established that 5-OH-TMT activates the Dectin-1 pathway, we next sought to prove that this effect was mediated by a direct inhibition of HTRA2. Notably, siRNA-mediated silencing of *HTRA2* in epithelial cells phenocopied the effects of 5-OH-TMT. Pathway analysis confirmed that *HTRA2* knockdown was sufficient to enrich the C-type lectin receptor signaling pathway and upregulate *CLEC7A* (Fig. 7a-d). Moreover, silencing of *HTRA2* induced a broad mucosal defense program, with significant increases evident in the expression of genes essential for the tight junction barrier (*CLDN1*, *OCLN*), the mucus layer (*MUC2*), and intracellular pathogen sensing (*NOD2*) (Fig. 7e).

Taken together, these data established a clear mechanistic cascade whereby 5-OH-TMT directly inhibits HTRA2, which relieves a brake on the Dectin-1 signaling axis and unleashes a coordinated transcriptional program to enhance epithelial defense and resolve inflammation.

### HTRA2 inhibition is sufficient to mediate the protective effects of 5-OH-TMT

**Fig. 8:**
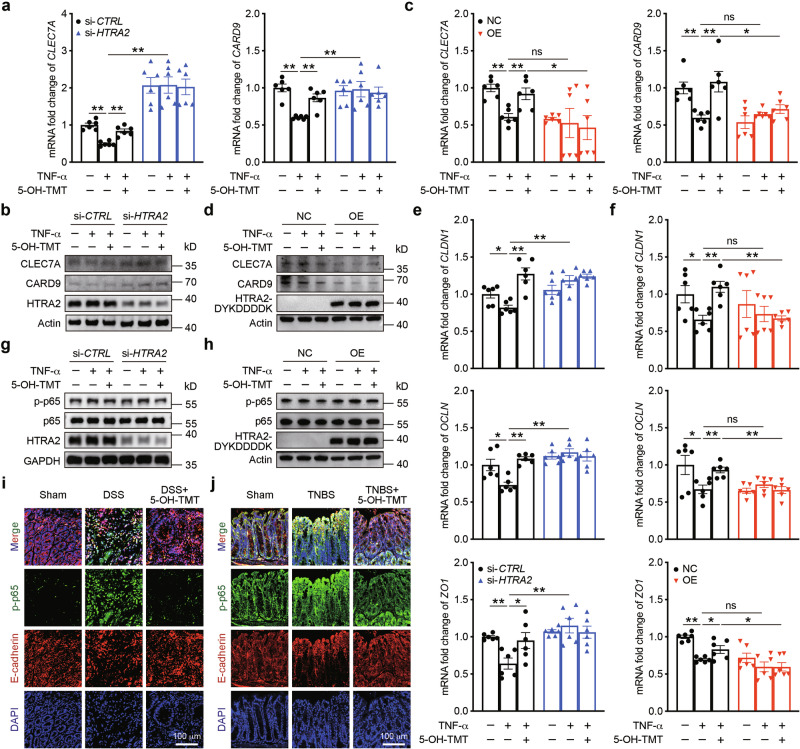
HTRA2 inhibition is sufficient to mediate the protective effects of 5-OH-TMT. **a-d** CLEC7A and CARD9 mRNA expression (**a**, **c**) and protein levels (**b**, **d**) in NCM460 cells infected with si-*CTRL* and si-*HTRA2* or NC and OE stimulated by TNF-*α* and treated with or without 5-OH-TMT. **e**, **f** Tight junction genes *CLDN1*, *OCLN*, *ZO1* mRNA expression in NCM460 cells infected with si-*CTRL* and si-*HTRA2* (**e**) or NC and OE (**f**) stimulated by TNF-*α* and treated with or without 5-OH-TMT. **g**, **h** Phosphorylation levels of p65 in HTRA2 knockdown (**g**) or overexpression (**h**) NCM460 cells. **i**, **j** Colon tissue sections from DSS-induced (**i**) or TNBS-indued (**j**) mice treated with 30 mg/kg 5-OH-TMT or vehicle were immune-stained for p-p65 (*green*) together with E-cadherin (*red*) prior to analysis by confocal microscopy. Scale bars, 100 μm. Data are represented as mean ± SEM. *n* = 6. The *P* values are determined by Tukey’s multiple-comparison test for **a**, **c**, **e**, and **f**. *P < 0.05, **P < 0.01, ns, not significant.

We further elucidated the role of HTRA2 as a critical upstream target of 5-OH-TMT by conducting both loss-of-function and gain-of-function experiments. siRNA-mediated knockdown of HTRA2 mimicked the effect of 5-OH-TMT and resulted in a significant upregulation of CLEC7A and CARD9 expression at both the mRNA (Fig. 8a) and protein levels (Fig. 8b). In contrast, overexpression of HTRA2 not only failed to increase the expression of these genes but it antagonized the upregulation of CLEC7A and CARD9 by 5-OH-TMT (Fig. 8c, d). Furthermore, *HTRA2* knockdown recapitulated the barrier-enhancing effects of 5-OH-TMT by significantly increasing the expression of key tight junction genes (*CLDN1*, *OCLN*, and *ZO1)* (Fig. 8e), whereas HTRA2 overexpression blocked the 5-OH-TMT-mediated enhancement of these junctional components (Fig. 8f). The modulation of HTRA2 also affected the expression of antimicrobial peptides (Fig. S11).

Having confirmed that HTRA2 inhibition drives the protective and pro-resolving pathways, we next sought to link HTRA2 to the suppression of pro-inflammatory signaling. Both direct treatment with 5-OH-TMT and siRNA-mediated silencing of *HTRA2* resulted in significant in vitro suppression of the phosphorylation of the NF-κB p65 subunit (Fig. 8g), whereas HTRA2 overexpression did not achieve this inhibition (Fig. 8h). In vivo validation of this pivotal anti-inflammatory mechanism confirmed that in vivo, where 5-OH-TMT treatment markedly reduced p-p65 levels in the colonic epithelium of mice with colitis (Fig. 8i, j). Further exploration of the proapoptotic function of HTRA2 in our colitis mouse models revealed that 5-OH-TMT treatment significantly reduced the number of TUNEL-positive cells in the colonic mucosa (Fig. S12), demonstrating its antiapoptotic effect under inflammatory conditions.

Collectively, these results provided a complete mechanism of action whereby 5-OH-TMT mitigates colitis by directly binding and inhibiting HTRA2. This single molecular event orchestrates a dual therapeutic response by simultaneously activating the protective Dectin-1/CARD9 pathway to restore intestinal homeostasis while suppressing pro-inflammatory NF-κB signaling.

## Discussion

In this study, we provide a comprehensive preclinical validation of 5-OH-TMT as a novel therapeutic agent for the treatment of inflammatory bowel disease, and we elucidate its underlying molecular mechanism of action. We demonstrate that 5-OH-TMT confers potent protection in two distinct mouse models of colitis via an effect driven by a dual mechanism involving the suppression of pro-inflammatory signaling and the active restoration of intestinal epithelial barrier integrity. The most significant finding of our study is the identification of the mitochondrial serine protease HTRA2 as the direct molecular target of 5-OH-TMT. We establish a clear mechanistic cascade in which the inhibition of HTRA2 by 5-OH-TMT relieves a repressive signal on the Dectin-1/CARD9 pathway, thereby unleashing a coordinated mucosal defense program that collectively mitigates colitis.

Our in vivo experiments robustly establish the therapeutic potential of 5-OH-TMT by demonstrating significant efficacy in both the innate immunity-driven DSS model and the T-cell–mediated TNBS model of colitis. Treatment resulted in marked improvements in clinical parameters, including reduced weight loss and disease activity scores, as substantiated by histological evidence of preserved mucosal architecture. We propose that this broad effectiveness stems from a reinforcement of the intestinal epithelial barrier via the HTRA2–Dectin-1 signaling axis. This barrier is a central pathological nexus in both models, so limiting the translocation of microbial products in DSS colitis and restricting hapten penetration in TNBS colitis by barrier fortification effectively dampens the subsequent inflammatory cascade.

This mechanism is further supported by the ability of 5-OH-TMT to promote a profound remodeling of the cytokine network by suppressing pro-inflammatory mediators (TNF-*α*, IL-6, IL-1*β*) while boosting anti-inflammatory IL-10. Thus, by targeting the fundamental vulnerability of barrier dysfunction, the HTRA2–Dectin-1 pathway ultimately uncouples epithelial injury from downstream inflammation. This effectively rebalances the local immune environment, irrespective of whether the pathology is primarily innate or adaptive, and highlights the efficacy of 5-OH-TMT as a broadly applicable therapeutic drug for IBD.

A critical component of IBD pathogenesis is the failure of the intestinal barrier [29, 30], and our data clearly show that 5-OH-TMT directly counteracts this defect. The restoration of the expression and proper apical localization of key tight junction proteins, including CLDN1, OCLN, and ZO-1, provides a direct molecular explanation for how 5-OH-TMT reinforces barrier function. This action likely limits the translocation of luminal antigens, thereby breaking the vicious cycle of inflammation. Furthermore, the upregulation of antimicrobial peptides suggests that 5-OH-TMT enhances the chemical barrier of the epithelium and adds another layer to its mucosal protective effects. Together, these findings position 5-OH-TMT as a multi-faceted agent that can dampen inflammation while also actively promoting mucosal healing.

The central finding of our work is the successful deorphanization of the therapeutic action of 5-OH-TMT by identifying HTRA2 as its direct target. Our use of an unbiased TRAP screening approach, followed by orthogonal validation with CETSA, DARTS, and MST, enabled us to build a compelling case for a direct and specific physical interaction. The selectivity for HTRA2 over its homolog HTRA1 further underscores the specificity of this engagement. Our molecular docking simulations predicted binding to the catalytic pocket, while the TRAP data identified a functionally important protease domain loop (aa 234–247) whose accessibility was altered. These results are complementary rather than contradictory and suggest a model in which 5-OH-TMT binds within the catalytic site (Asn181, Lys395) to induce a conformational change in the adjacent substrate-binding loop, thereby inhibiting protease function. This detailed insight into the drug–target interface provides a solid foundation for future structure-based drug optimization.

Our identification of the 5-OH-TMT target enabled us to follow the novel downstream pathway linking HTRA2 inhibition to intestinal protection. Our transcriptomic analysis unexpectedly pointed toward the C-type lectin receptor signaling pathway, specifically revealing an upregulation of Dectin-1 (CLEC7A) and its key adaptor, CARD9. Dectin-1 is a pattern recognition receptor renowned for its role in antifungal immunity [31–34], but it is now increasingly recognized as a critical regulator of intestinal homeostasis and protection against colitis [35–40]. The activation of the Dectin-1/CARD9 axis is known to regulate inflammatory responses [41]; thus, our discovery that 5-OH-TMT activates this pathway in vivo establishes a previously unknown connection between HTRA2 and C-type lectin receptor signaling.

Our *HTRA2* silencing experiments revealed the final and most definitive piece of our mechanistic puzzle. A particularly remarkable finding was that siRNA-mediated knockdown of *HTRA2* phenocopied the key therapeutic effects of 5-OH-TMT including the upregulation of CLEC7A/CARD9, the enhancement of tight junction gene expression, and the suppression of NF-κB p65 phosphorylation. This phenocopying provides unequivocal evidence that HTRA2 is the critical upstream regulator and confirms that HTRA2 normally functions as a negative regulator, or a brake, on this protective mucosal defense pathway. By inhibiting HTRA2, 5-OH-TMT effectively “cuts the brake lines,” allowing the Dectin-1/CARD9 axis to engage and restore intestinal homeostasis. Thus, this single molecular event orchestrates the dual therapeutic outcomes observed throughout our study.

Zhang et al. have also identified HTRA2 inhibition as a potent therapeutic strategy for the treatment of colitis by demonstrating that pharmacological blockade of its protease activity reduces inflammation and restores intestinal barrier function [42]. However, their postulated underlying mechanism differs fundamentally from the mechanism documented in the present study. Zhang et al. posited that HTRA2 directly promotes pro-inflammatory necroptosis and that its inhibition is protective because it prevents the operation of this deleterious cell death pathway. In contrast, our work elucidates a novel role for HTRA2 as a negative regulator of a protective mucosal defense program, whereby its inhibition activates the Dectin-1 signaling axis to promote intestinal homeostasis. Although both studies have arrived at differing mechanistic conclusions, both investigations collectively establish HTRA2 as a critical and multifaceted therapeutic target for IBD.

A particularly intriguing finding of our study is the mechanistic link between the inhibition of HTRA2, a mitochondrial protease, and the transcriptional upregulation of the cell-surface receptor Dectin-1. While mitochondrial retrograde signaling offers one possible explanation, an alternative and compelling hypothesis invokes the involvement of a more direct regulatory axis. We propose that the function of HTRA2 extends beyond mitochondrial quality control to include the regulation of a key cytoplasmic signaling intermediate that governs CLEC7A gene expression. In this model, the constitutive protease activity of HTRA2 may serve to cleave and degrade a putative activator of Dectin-1 transcription, thereby maintaining its low basal expression. Upon inhibition of HTRA2 by 5-OH-TMT, this activator is no longer degraded and instead stabilizes and accumulates. Subsequently, this stabilized factor could translocate to the nucleus and drive the observed upregulation of Dectin-1 transcription. This mechanism is plausible given that HTRA2 is known to be released from the mitochondria under certain stress conditions and can cleave cytoplasmic substrates. Therefore, identifying this putative signaling intermediate and confirming its cleavage by HTRA2 represents a high-priority direction for our future work, as this could definitively establish this novel regulatory pathway.

While our findings are compelling, several limitations should be acknowledged. First, the absence of a direct comparison between 5-OH-TMT and its parent compound, bufotenine, reflects a deliberate, hypothesis-driven design. Quaternization of the terminal nitrogen, which introduces a permanent positive charge, is a modification specifically intended to prevent blood–brain barrier penetration and enhance metabolic stability. By converting a centrally active hallucinogen into a peripherally restricted agent, 5-OH-TMT is best evaluated within a distinct pharmacological framework aimed at mitigating central nervous system liabilities rather than as a direct bufotenine analog.

Regarding our study’s experimental scope, the work was conducted in acute models of colitis; therefore, the efficacy of 5-OH-TMT in chronic relapsing conditions remains to be determined. Additionally, while we have established a clear functional link in which HTRA2 regulates Dectin-1, the precise molecular mechanism by which the HTRA2 protease controls this pathway is still unknown. Finally, because our analysis focused on epithelial and whole-tissue responses, the cell-type–specific effects of 5-OH-TMT on resident immune cells within the lamina propria require further exploration. Addressing these gaps, specifically the transition to chronic models and the dissection of molecular and cellular mechanisms, will be crucial for full translation of the therapeutic potential of this peripherally restricted scaffold.

In summary, our study identifies 5-OH-TMT as a potent therapeutic agent for the treatment of experimental colitis. We have elucidated a complete mechanism of action by demonstrating that 5-OH-TMT directly binds to and inhibits the mitochondrial protease HTRA2. This inhibition triggers a dual therapeutic program involving the suppression of pro-inflammatory NF-κB signaling and the simultaneous activation of the protective Dectin-1/CARD9 mucosal defense pathway. By uncovering this novel HTRA2–Dectin-1 axis, our work provides a strong rationale for the clinical development of 5-OH-TMT for IBD, while also validating HTRA2 as a promising and druggable new target for modulating intestinal inflammation and promoting mucosal healing.

## Supplementary information

Supplementary information

## Data Availability

The accession number for the RNA-seq reported in this study in GEO is GSE302465 https://www.ncbi.nlm.nih.gov/geo/query/acc.cgi?acc=GSE302465.
