# Supplementary material for: 5-OH-TMT mitigates colitis through HTRA2 binding–mediated activation of the Dectin-1 signaling pathway
Source: Acta Pharmacol Sin. 2026 Apr 2;47(8):2182–95. doi: 10.1038/s41401-026-01782-0 (PMC13389218; doi:10.1038/s41401-026-01782-0)
Supplement: Supplementary file 1 — Supplementary information [file 41401_2026_1782_MOESM1_ESM.docx]

**Supplementary Files**

**Supplementary Figures**

Figure S1. 5-OH-TMT reduces pro-inflammatory cytokine production in DSS-induced colitis.

Figure S2. 5-OH-TMT increases expression of antimicrobial peptides in DSS- and TNBS-induced colitis.

Figure S3. Effect of 5-OH-TMT on NCM460 cell viability.

Figure S4. 5-OH-TMT does not affect HTRA1 protease sensitivity.

Figure S5. Molecular docking analysis of 5-OH-TMT binding to HTRA2.

Figure S6. The interaction of 5-OH-TMT with HTRA2 mutants were measured by MST.

Figure S7. Analysis of the combined area of 5-OH-TMT and HTRA2 via TRAP experiment.

Figure S8. Overlap of differentially expressed genes in response to TNF-*α* and 5-OH-TMT treatment.

Figure S9. **GSEA of the C-type lectin receptor signaling pathway.**

Figure S10. 5-OH-TMT upregulates *Clec7a* and *Card9* expression in DSS- but not TNBS-induced colitis.

Figure S11. HTRA2 regulates antimicrobial peptide expression.

Figure S12. 5-OH-TMT attenuates colonic apoptosis in murine colitis models.

**Supplementary Table**

Table S1. Primers for quantitative PCR analysis.


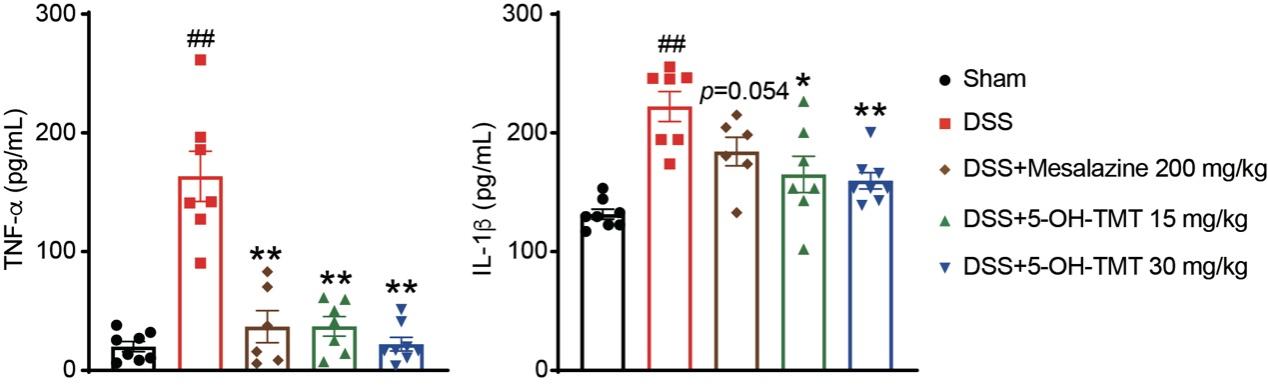


**Fig. S1. 5-OH-TMT reduces pro-inflammatory cytokine production in DSS-induced colitis.** TNF-*α* and IL-1*β* levels in serum from sham, DSS, DSS+mesalazine (200 mg/kg), DSS+5-OH-TMT (15 mg/kg), and DSS+5-OH-TMT (30 mg/kg) groups, as measured by ELISA (*n* = 6-8 per group). Data are presented as mean ± SEM. *P* values are determined by Tukey multiple comparison test. ^##^*P* < 0.01 vs. Sham; ^**^*P* < 0.01, ^*^*P* < 0.05 vs. DSS.


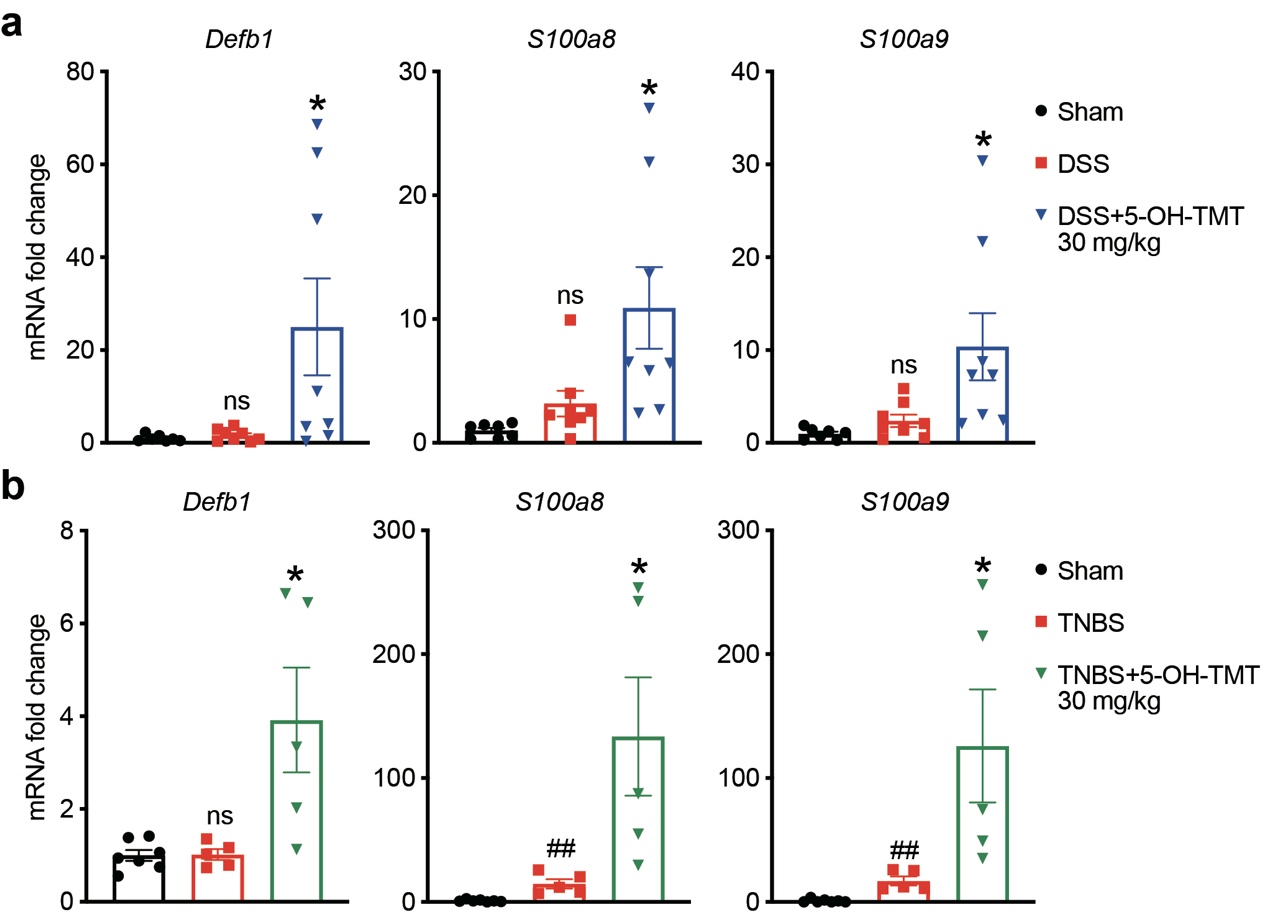


**Fig. S2. 5-OH-TMT increases expression of antimicrobial peptides in DSS- and TNBS-induced colitis.** **a** Relative mRNA expression of *Defb1*, *S100a8*, and *S100a9* in colon tissues from sham, DSS, and DSS+5-OH-TMT (30 mg/kg) groups, as determined by qPCR (*n* = 8 per group). **b** Relative mRNA expression of *Defb1*, *S100a8*, and *S100a9* in colon tissues from sham, TNBS, and TNBS+5-OH-TMT (30 mg/kg) groups, as determined by qPCR (*n* = 5-6 per group). Data are presented as mean ± SEM. *P* values are determined by two-tailed Student’s *t*-test. ^##^*P* < 0.01 vs. Sham; ns, not significant; ^*^*P* < 0.05 vs. DSS or TNBS.


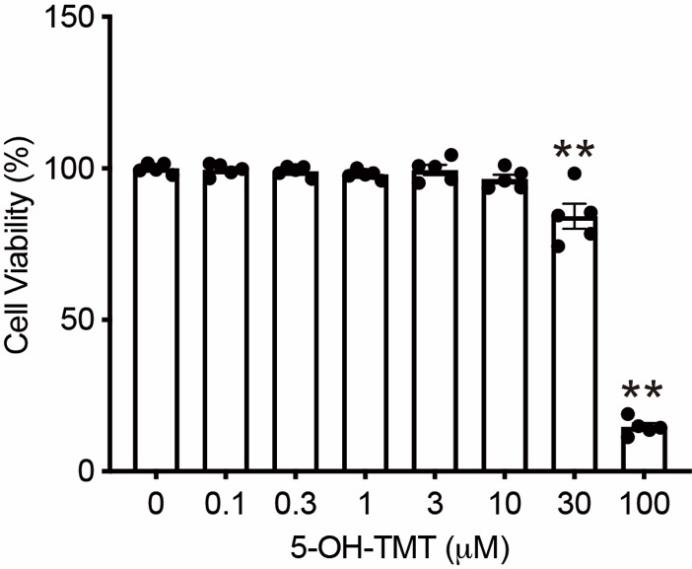


**Fig. S3.** **Effect of 5-OH-TMT on NCM460 cell viability.** Cell viability of NCM460 cells treated with increasing concentrations of 5-OH-TMT (0–100 μM) for 96 hours, as assessed by CCK-8 assay (*n* = 5 per group). Data are presented as mean ± SEM. *P* values are determined by two-tailed Student’s *t*-test. ^**^*P* < 0.01 vs. 0 μM.


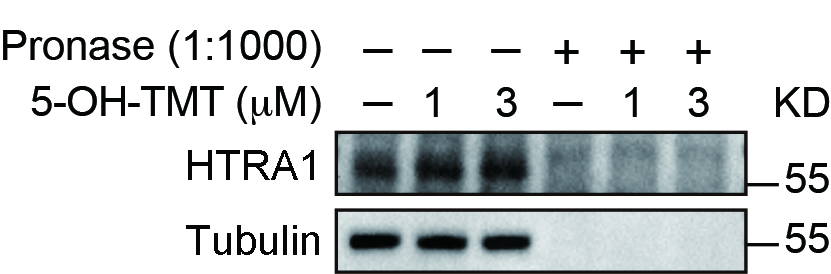


**Fig. S4.** **5-OH-TMT does not affect HTRA1 protease sensitivity.** Western blot analysis of HTRA1 protein levels in the presence or absence of pronase (1:1000) and 5-OH-TMT (1 or 3 μM).


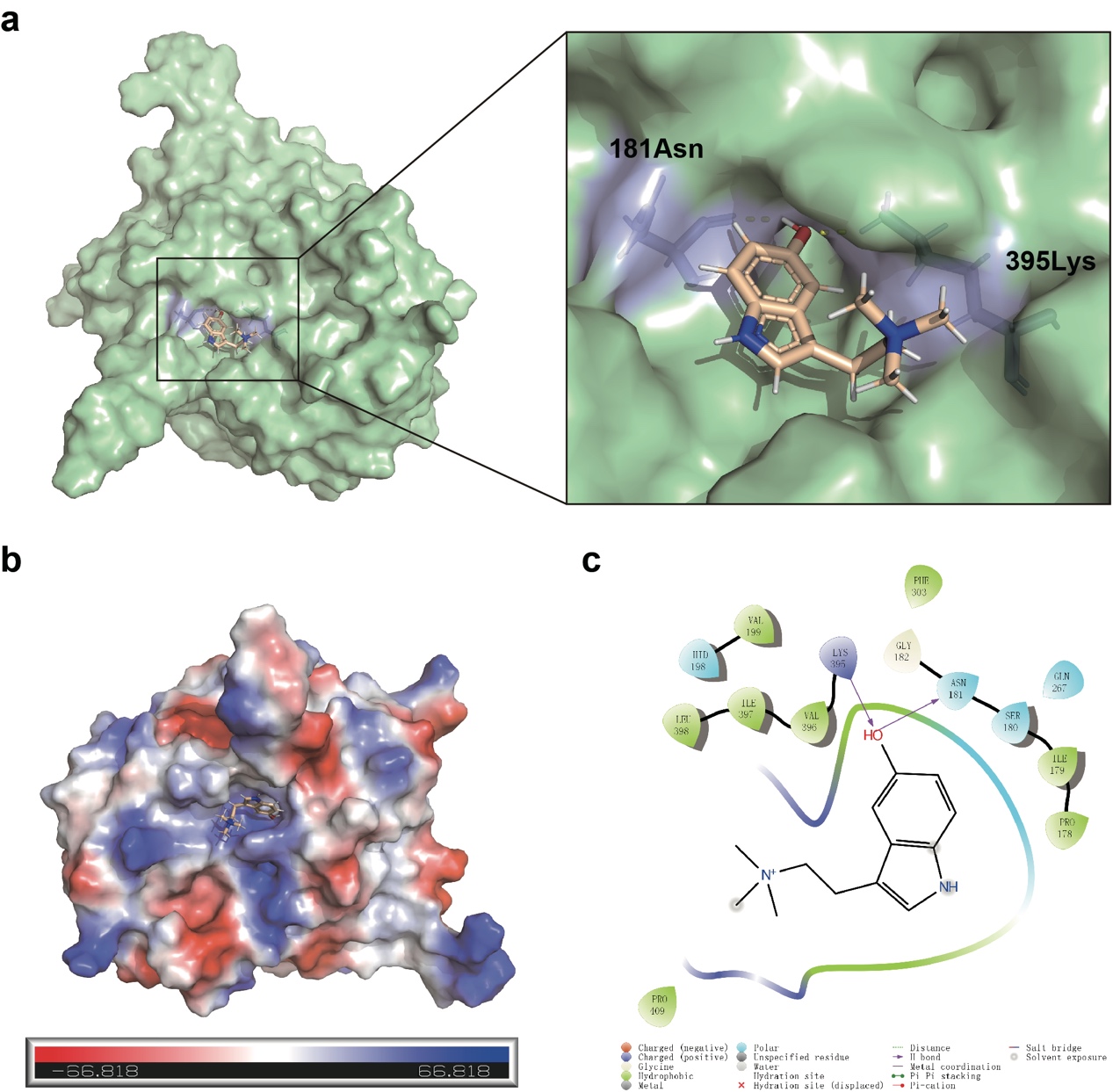


**Fig. S5.** **Molecular docking analysis of 5-OH-TMT binding to HTRA2.** **a** Predicted binding mode of 5-OH-TMT (shown in stick representation) within the active site of HTRA2, with a close-up view highlighting key interacting residues Asn181 and Lys395. **b** Electrostatic surface potential map of HTRA2 showing the location of 5-OH-TMT within the binding pocket. **c** Schematic diagram of the predicted interactions between 5-OH-TMT and amino acid residues in the HTRA2 binding pocket, including hydrogen bonds and hydrophobic contacts.


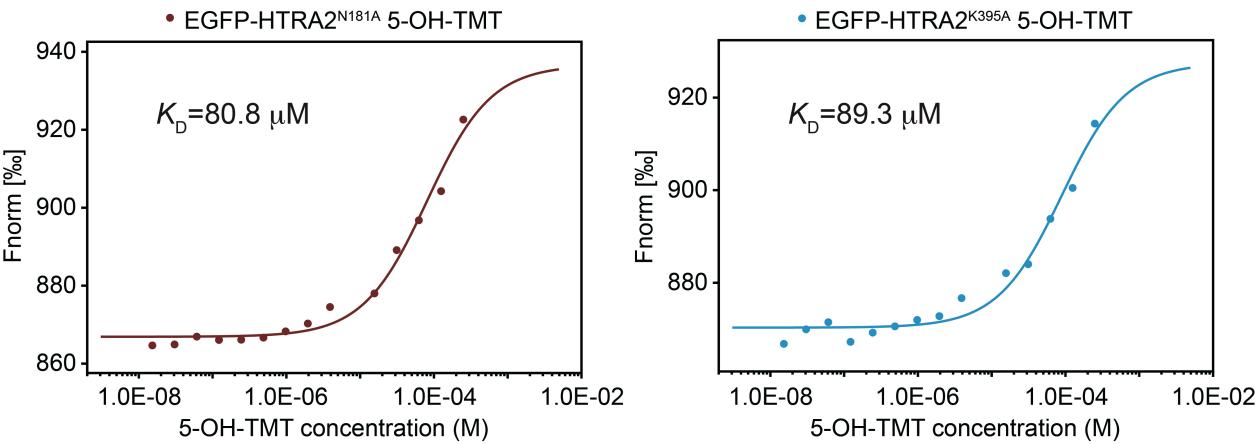


**Fig. S6. The interaction of 5-OH-TMT with HTRA2 mutants were measured by MST.**

**
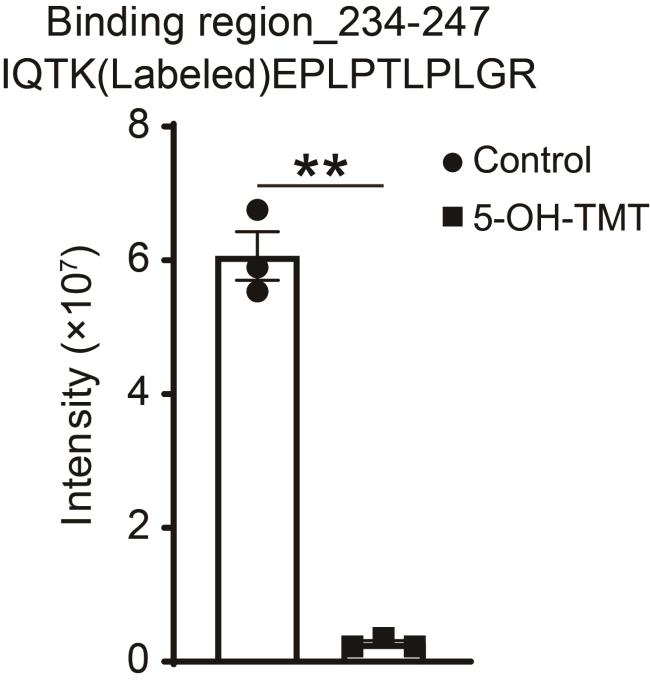
**

**Fig. S7. Analysis of the combined area of 5-OH-TMT and HTRA2 via TRAP experiment.** Data are presented as mean ± SEM. *P* values are determined by two-tailed Student’s *t*-test. ^**^*P* < 0.01 vs. control.


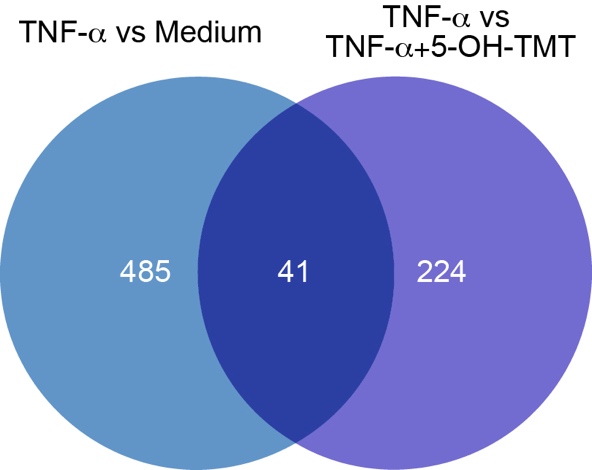


**Fig. S8.** **Overlap of differentially expressed genes in response to TNF-*α* and 5-OH-TMT treatment.** Venn diagram showing the number of differentially expressed genes (DEGs) identified in TNF-*α* vs medium (|Log₂(Fold Change)|>1, *P* < 0.05) and TNF-*α* vs TNF-*α* plus 5-OH-TMT groups (|Log₂(Fold Change)|>1, *P* < 0.05).


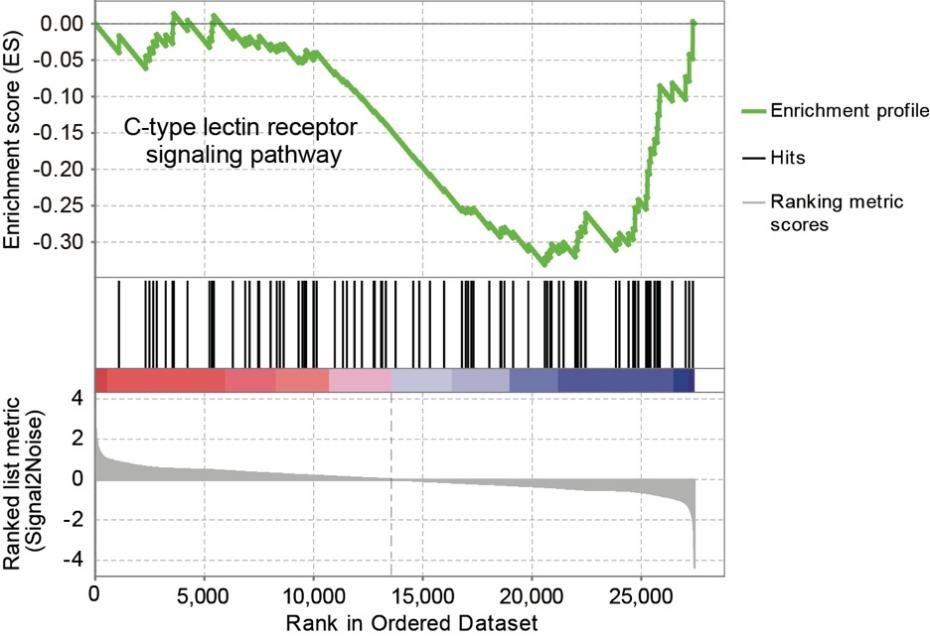


**Fig. S9. GSEA of the C-type lectin receptor signaling pathway.** GSEA plot showing negative enrichment of the C-type lectin receptor signaling pathway in NCM460 cells following 5-OH-TMT treatment. The enrichment score (ES) is plotted against gene rank in the ordered dataset. Black vertical lines indicate the positions of pathway genes within the ranked list.


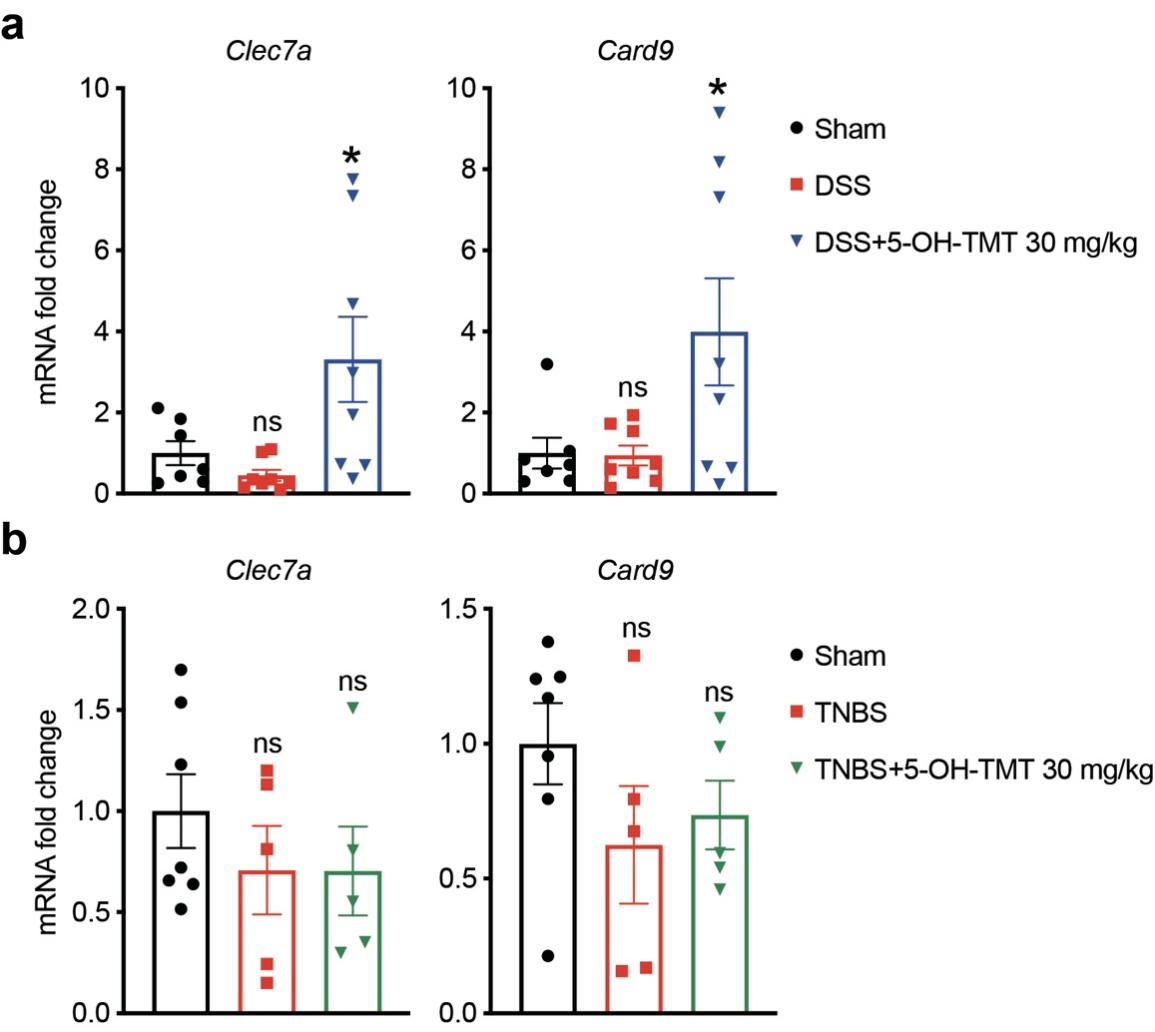


**Fig. S10. 5-OH-TMT upregulates *Clec7a* and *Card9* expression in DSS- but not TNBS-induced colitis. a** Relative mRNA expression of *Clec7a* and *Card9* in colon tissues from sham, DSS, and DSS+5-OH-TMT (30 mg/kg) groups, as determined by qPCR (*n* = 8 per group). **b** Relative mRNA expression of *Clec7a* and *Card9* in colon tissues from sham, TNBS, and TNBS+5-OH-TMT (30 mg/kg) groups, as determined by qPCR (*n* = 5-6 per group). Data are presented as mean ± SEM. *P* values are determined by two-tailed Student’s *t*-test. ^*^*P* < 0.05 vs. DSS or TNBS group; ns, not significant.


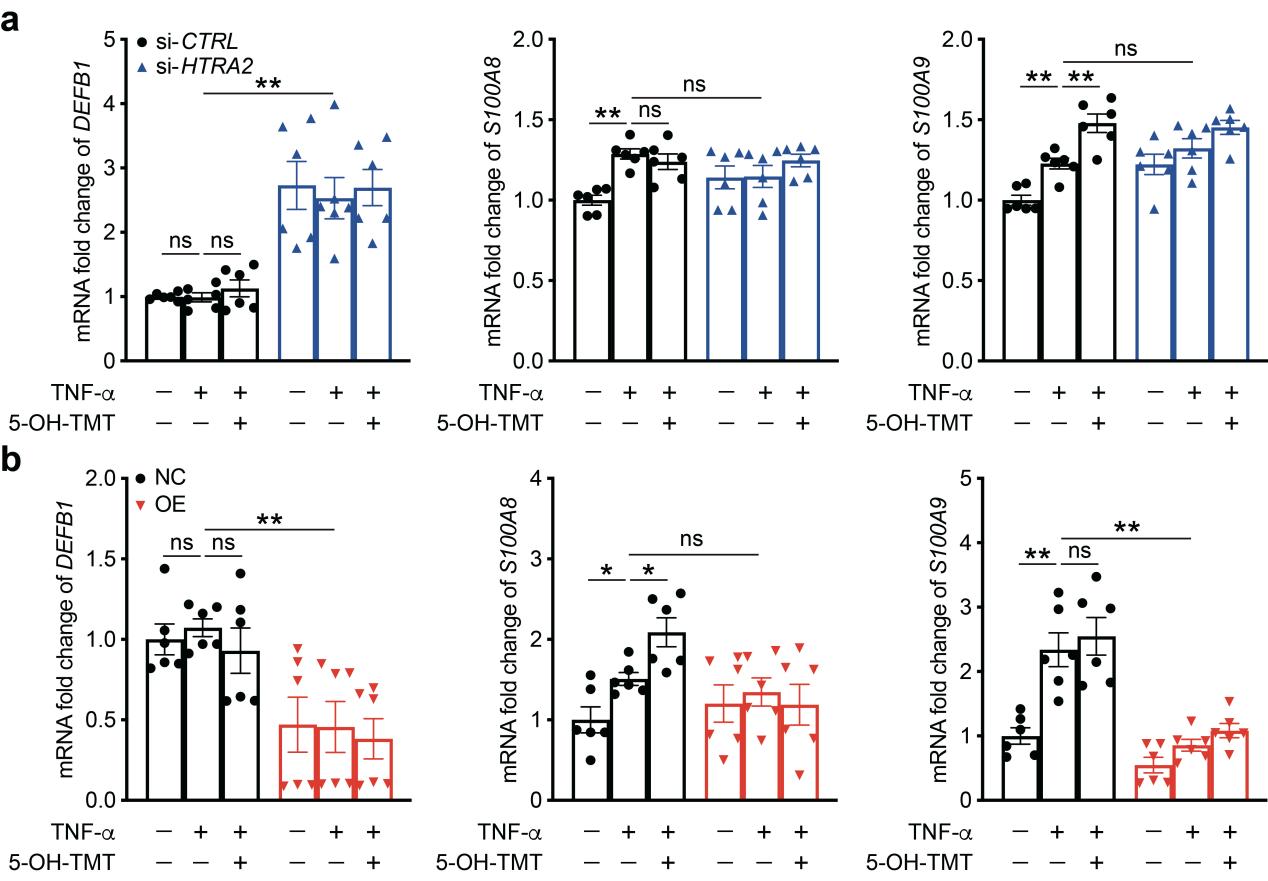


**Fig. S11.** **HTRA2 regulates antimicrobial peptide expression. a**,**b** Antimicrobial peptides genes *DEFB1*, *S100A8*, *S100A9* mRNA expression in NCM460 cells infected with si-*CTRL* and si-*HTRA2* (**a**) or NC and OE (**b**) stimulated by TNF-*α* and treated with or without 5-OH-TMT. Data are represented as mean ± SEM, *n* = 6. The *P* values are determined by Tukey’s multiple-comparison test. ***P* < 0.01, **P* < 0.05, ns, not significant.


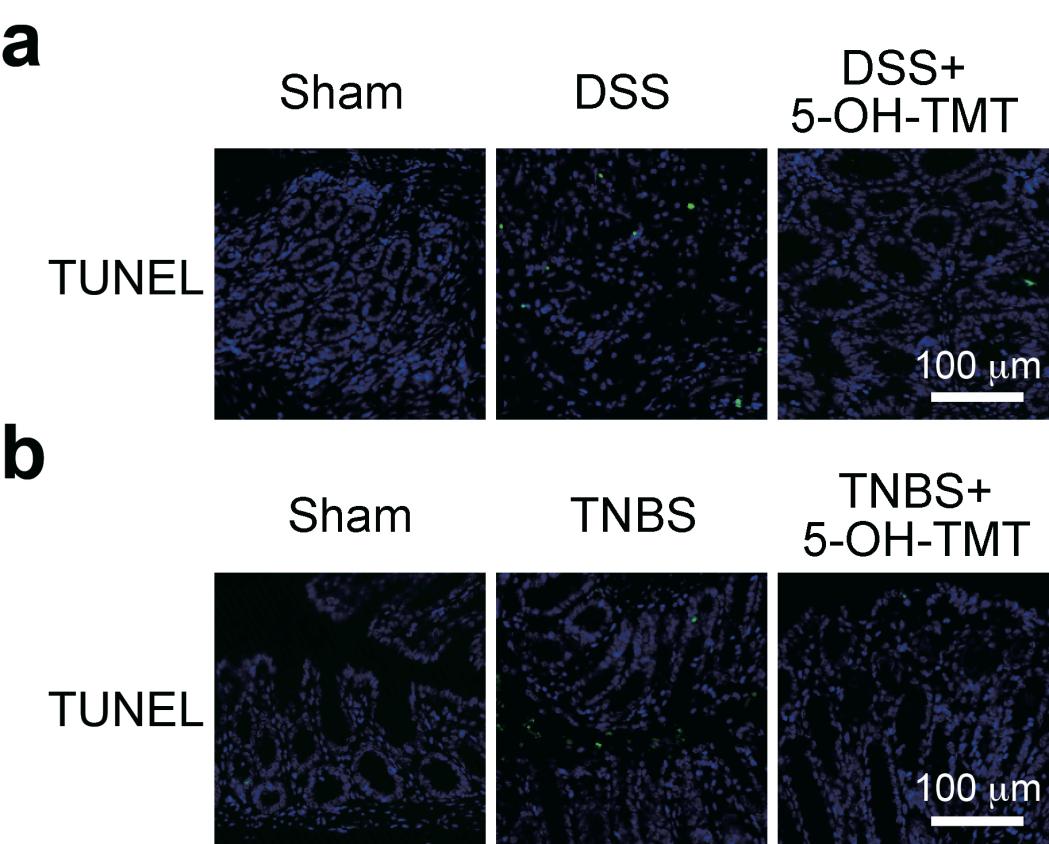


**Fig. S12. 5-OH-TMT attenuates colonic apoptosis in murine colitis models. a,b** Colon tissue sections from DSS-induced (**a**) or TNBS-induced (**b**) mice treated with 30 mg/kg 5-OH-TMT or vehicle were immune-stained for TUNEL (*green*) prior to analysis by confocal microscopy. Scale bars, 100 μm.

**Table S1**. Primers for quantitative PCR analysis.

| **Primers** | **Sequences（5' to 3'）** | |
| --- | --- | --- |
| mouse-*Gapdh-F* | GGTTGTCTCCTGCGACTTCA | |
| mouse*-Gapdh-R* | TGGTCCAGGGTTTCTTACTCC | |
| mouse-*Tnfa-F* | GACGTGGAACTGGCAGAAGAG | |
| mouse-*Tnfa-R* | TTGGTGGTTTGTGAGTGTGAG | |
| mouse-*Il6-F* | CAAAGCCAGAGTCCTTCAGAG | |
| mouse-*Il6-R* | GTCCTTAGCCACTCCTTCTG | |
| mouse-*Il1b-F* | ACGGACCCCAAAAGATGAAG | |
| mouse-*Il1b-R* | TTCTCCACAGCCACAATGAG | |
| mouse-*Il10-F* | ACCTGCTTTCCCCAAAACGAA | |
| mouse-*Il10-R* | TGAGAGAAGTCGCACTGAGTC | |
| mouse-*Cldn1-F* | AGGTCTGGCGACATTAGTGG | |
| mouse-*Cldn1-R* | CGTGGTGTTGGGTAAGAGGT | |
| mouse-*Ocln-F* | ACACTTGCTTGGGACAGAGG | |
| mouse-*Ocln-R* | AAGGAAGCGATGAAGCAGAA | |
| mouse-*Zo1-F* | GACCTTGATTTGCATGACGA | |
| mouse-*Zo1-R* | AGGACCGTGTAATGGCAGAC |  |
| mouse-*Clec7a-F* | GACTTCAGCACTCAAGACATCC |  |
| mouse-*Clec7a-R* | TTGTGTCGCCAAAATGCTAGG |  |
| mouse-*Card9-F* | TCCAGACGGAGAGCCGATTA |  |
| mouse-*Card9-R* | CCTGGGTGAACTGTCTTCCAA |  |
| mouse-*Defb1-F* | AGGTGTTGGCATTCTCACAAG |  |
| mouse-*Defb1-R* | GCTTATCTGGTTTACAGGTTCCC |  |
| mouse-*S100a8-F* | AAATCACCATGCCCTCTACAAG |  |
| mouse-*S100a8-R* | CCCACTTTTATCACCATCGCAA |  |
| mouse-*S100a9-F* | ATACTCTAGGAAGGAAGGACACC |  |
| mouse-*S100a9-R* | TCCATGATGTCATTTATGAGGGC |  |
| human-*ACTIN*-F | ATTGGCAATGAGCGGTTC | |
| human-*ACTIN*-R | GGATGCCACAGGACTCCAT | |
| human-*CLEC7A*-F | TGGGAGGATGGATCAACATT | |
| human-*CLEC7A*-R | TGGGTTTTCTTGGGTAGCTG | |
| human-*CARD9*-F | ATGTCGGACTACGAGAACGAT | |
| human-*CARD9*-R | TGATGCGTGAGGGGTCGAT | |
| human-*CLDN1*-F | CCTCCTGGGAGTGATAGCAAT | |
| human-*CLDN1*-R | GGCAACTAAAATAGCCAGACCT | |
| human-*OCLN*-F | ACAAGCGGTTTTATCCAGAGTC | |
| human-*OCLN*-R | GTCATCCACAGGCGAAGTTAAT | |
| human-*ZO1*-F | CAACATACAGTGACGCTTCACA | |
| human-*ZO1*-R | CACTATTGACGTTTCCCCACTC | |
| human-*DEFB1*-F | ATGAGAACTTCCTACCTTCTGCT | |
| human-*DEFB1*-R | TCTGTAACAGGTGCCTTGAATTT | |
| human-*S100A8*-F | ATTTTGGGGAGACCTGGTGG | |
| human-*S100A8*-R | GCTTCCAGGCCCACCTTTAT | |
| human-*S100A9*-F | GCTTCTCGGCTTGGTAGGAG | |
| human-*S100A9*-R | GCTTCTCGGCTTGGTAGGAG | |
